# Surgical repair of an obstructed mixed‐type total anomalous pulmonary venous connection

**DOI:** 10.1002/ccr3.6747

**Published:** 2022-12-18

**Authors:** Reem Shammout, Doaa Alkhadraa, Sidra Alkadi, Alwaleed Al‐Dairy

**Affiliations:** ^1^ Medical student at Faculty of Medicine Damascus University Damascus Syria; ^2^ Cardiac Surgery at Faculty of Medicine Damascus University Damascus Syria

**Keywords:** patent foramen ovale, pulmonary venous obstruction, Total anomalous pulmonary venous connection, ventricular septal defect

## Abstract

Total anomalous pulmonary venous connection is a rare congenital anomaly and has four anatomical subtypes of which the mixed type represents diagnostic and therapeutic challenge. When associated with obstruction, however, urgent surgical repair is needed. Herein, we present a rare case of obstructed mixed type total anomalous pulmonary venous connection with successful surgical repair.

## Abstract 2

Total anomalous pulmonary venous connection has four main anatomical types. When associated with obstruction, however, it needs urgent surgical repair. Accurate preoperative evaluation is critical for achieving favorable outcomes.

## 1 INTRODUCTION

Total anomalous pulmonary venous connection (TAPVC) is one of the rarest congenital cardiac diseases with 7 per 100,000 neonates' approximate incidence.^1^, ^2^ It accounts for about 1% of all congenital heart diseases.^3^ In this anomaly, all the pulmonary veins drain into right atrium (or one of its tributaries) instead of the normal drainage into left atrium.^4^ Anatomically, TAPVC can be divided into 4 major types depending on the level of pulmonary veins' drainage: supracardiac (into the innominate vein or right superior vena cava), cardiac (into the coronary sinus or directly to the right atrium), infracardiac (most commonly into the portal vein or into the ductus venosus, hepatic vein, or directly into the infradiaphragmatic inferior vena cava), and mixed (in which there is a combination between types above).^1^, ^2^, ^3^, ^4^ The separation of both pulmonary and systemic circulations in TAPVC makes the presence of right‐to‐left shunt obligatory for survival, and any restriction in this communication represents an “obstruction.” The obstruction may be at any level of the drainage and is encountered in about 25%–50% of TAPVC patients. The degree and level of obstruction are remarkable factors affecting the pathophysiology and presentation.^5^, ^6^ In the presence of obstruction, the patient is usually symptomatic in the neonatal period, symptoms progressively worsen, and the neonate may present with profound acidosis and hypoxemia despite the aggressive management and oxygen supplement.^4^ Transthoracic echocardiography (TTE) is the main diagnostic tool; however, computed tomographic angiography (CTA) plays a major role in such anomalies and adds an important anatomic details, especially regarding the site of any obstruction.^4^ Herein, we report a rare case of an obstructed mixed‐type TAPVC in a neonate in association with large PDA and VSD in whom successful surgical repair was performed.

## 2 CASE PRESENTATION

A 7‐day‐old boy neonate presented to our hospital with cyanosis and tachypnea since birth.

**FIGURE 1:**
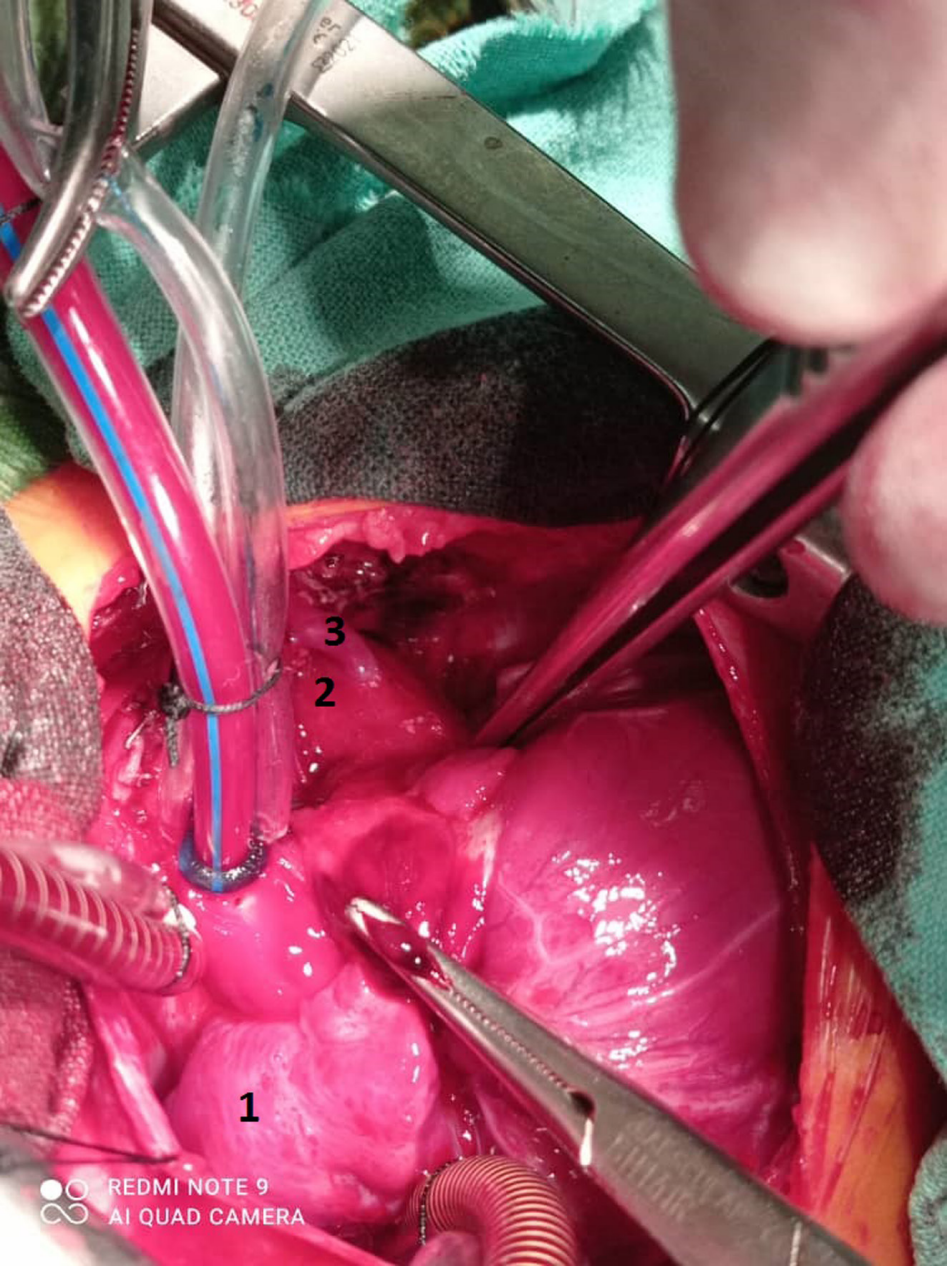
Intraoperative image showing the vertical vein with its connection to the left innominate vein. 1: Right atrium, 2: vertical vein, 3: the small branch that was connecting the vertical vein with the left innominate vein

TTE revealed mixed type TAPVC, small patent foramen ovale (PFO), a large VSD (10 mm), a large patent ductus arteriosus (PDA) with a right to left shunt, and severe pulmonary hypertension (PH). CTA was performed and confirmed the diagnosis of a mixed type TAPVC (the common pulmonary confluent was draining both into the coronary sinus and the left innominate vein through a vertical vein) with an obstruction. The obstruction level was at the connection of the vertical vein with the left innominate vein. On the 10th day of life, the neonate developed a sudden cardiac arrest and underwent cardiopulmonary resuscitation with rapid response without the need for mechanical ventilation. The patient was scheduled for urgent surgical repair. The operation was performed through median sternotomy. A large vertical vein was seen on the left side outside the pericardium and connecting with a very small vein to the left innominate vein (representing the site of obstruction) (Figure 1). The vertical vein was dissected and controlled. The pericardium was opened, and a large PDA was controlled and closed by metal clips. Complete cardiopulmonary bypass (CPB) was prepared with bicaval cannulation, the vertical vein was closed at its junction with the left innominate vein, and the heart was arrested by antegrade cold blood cardioplegic solution. The common pulmonary confluent was seen behind the heart. The right atrium was opened parallel to the right atrioventricular groove. A small PFO was seen (representing another site of obstruction). The coronary sinus orifice was cut through to unroof the coronary sinus and establish a wide communication with the left atrial cavity after resecting the atrial septum. A large fresh autologous pericardial patch was used to baffle the created cavity toward the left atrial cavity. A large VSD was closed by a bovine pericardium patch with interrupted sutures. The remainder of the operation was completed uneventfully and the patient was weaned off the CPB easily. The patient suffered from atrial arrhythmias (atrial ectopic tachycardia) in the intensive care unit (ICU) on the second postoperative day and was managed appropriately. After 1 week on mechanical ventilation, the patient was extubated, and on the 15th postoperative day was discharged from the ICU. The patient was followed up for 6 months and was in very good general condition with significant improvement and weight gain.

## 3 DISCUSSION

The diagnosis of mixed type TAPVC and its surgical repair are challenging; however, it is the least common type of TAPVC, accounting for about 5% of cases.^3^ Our patient had mixed type of TAPVC (cardiac and supracardiac) associated with large PDA and VSD. There were two sites of obstruction in our patient, the first was at the connection of the vertical vein with the left innominate vein which was through a very small vein branch (Figure 1), and the second was due to the very small PFO. The majority of TAPVC cases are diagnosed by TTE which is the preferred diagnostic tool, but sometimes additional image modalities are required such as CTA, angiography via cardiac catheterization or MRI.^7^, ^8^ In our case, CTA was crucial to diagnose the site of obstruction. The main management of TAPVC patients is primary surgical repair; however, a transcatheter palliative shunt may be considered in patients with other important comorbidities such as prematurity, low birth weight, multisystem organ dysfunction, multiple congenital anomalies.^9^, ^10^ Our patient was managed surgically and did not need primary transcatheter palliation. It has been reported that the mortality of obstructed TAPVC had decreased over the eras from 42.1% in the 1970s to 7.4% after 2010. The worst prognosis is predicted in patients with pulmonary obstruction and suprasystemic PH preoperatively in which the patient might have been intubated. Patients who underwent emergent surgery with significant pulmonary vein obstruction still have the worst scenario despite the progress in the field of TAPVC surgery.^11^

## 4 CONCLUSION

Mixed type TAPVC represents diagnostic and therapeutic challenge in the field of congenital heart surgery. The best surgical outcomes in obstructed TAPVC cases need accurate preoperative evaluation and prompt intervention.

## AUTHOR CONTRIBUTIONS

**Reem Shammout**: Participated in writing the report and approved the final version. **Doaa Alkhadraa**: wrote and reviewed the successive versions and participated in their revisions. **Sidra Alkadi**: wrote and reviewed the successive versions and participated in their revisions. **Alwaleed Al‐Dairy**: Planned and performed the work leading to the report. Wrote and reviewed successive versions and participated in their revisions.

## FUNDING INFORMATION

There are no funding resources for writing this manuscript.

## CONFLICT OF INTEREST

The authors have no conflict of interest.

## ETHICAL APPROVAL

The manuscript was approved by ethics committee at Damascus University.

## CONSENT

Written informed consent was obtained from the patient's parents to publish this report in accordance with the journal's patient‐consent policy**.**

## AUTHOR’S DECLARATION

None of the authors listed in the manuscript are employed by a government agency that has a primary function other than research and/or education. Moreover, none of the authors are submitting this manuscript as an official representative or on behalf of the government.

## Data Availability

The data that support the findings of this study are available from the corresponding author, [A.A], upon reasonable request.
